# Isolation-by-environment as a driver of genetic differentiation among populations of the only broad-leaved evergreen shrub *Ammopiptanthus mongolicus* in Asian temperate deserts

**DOI:** 10.1038/s41598-019-48472-y

**Published:** 2019-08-19

**Authors:** Shan Jiang, Min-Xin Luo, Run-Hong Gao, Wei Zhang, Yong-Zhi Yang, Ying-Jie Li, Pei-Chun Liao

**Affiliations:** 1 College of Grassland, Resources and Environment, Inner Mongolia Agricultural University, Huhhot, 010010 China; 2 School of Life Science, National Taiwan Normal University, No. 88 Ting-Chow Rd., Sec. 4, Taipei, 116 Taiwan; 3 College of Forestry, Inner Mongolia Agricultural University, Huhhot, 010019 China; 4 College of Mechanical and Electrical Engineering, Inner Mongolia Agricultural University, Huhhot, 010018 China

**Keywords:** Ecological genetics, Structural variation

## Abstract

Whether the effect of migration-selection-drift equilibrium on population structure is governed by spatial or environmental differences is usually elucidated by isolation-by-distance (IBD), isolation-by-environment (IBE), and isolation-by-resistance (IBR) tests. The population structure of *Ammopiptanthus mongolicus*, a broad-leaved evergreen psammophyte in eastern Central Asia, was previously thought to follow an isolation by distance pattern. However, recent studies have emphasized the effects of environmental factors on its growth and distribution, suggesting an important influence of local adaptation on the genetic structure of the species. Using inter-simple sequence repeat (ISSR) markers, we verified the previously inferred low intra-population variation and high inter-population differentiation. However, in contrast to previous studies, the results of partial Mantel tests and a maximum likelihood population effects mixed model (MLPE) suggested that local climate differences, rather than geographic distances or resistance distances, are the main factor affecting population differentiation. Further analysis with removal of multicollinear climatic variables and univariate MLPE found that summer and winter precipitation were crucial for shaping the current population genetic structure. Since local precipitation is related to the regeneration, colonization, and overwintering survival of *A. mongolicus*, its influence on demographic change may explain its effect on the population genetic structure. In addition, precipitation is related to terrain despite westward decreases, which explains the independence of genetic difference and geographic distance. The identified role of IBE suggests that collecting germplasm resources from genetically differentiated populations could be a more effective strategy to preserve the overall genetic diversity of the species than the establishment of corridors to enhance gene flow among populations.

## Introduction

Random genetic drift, environment-leading selection, genetic draft (hitchhiking), and background selection may affect the genetic diversity of organisms^1^. Geographic distance and environmental difference are two key factors affecting genetic structure between populations^2^. The former is related to the interplay of genetic drift and movement, while the latter is usually related to the adaptability to environmental pressure^3^. In changeable environments, selection determines genetic diversity of adapted genes, and the genetic diversity of neutral genes would be also reduced by random associations with genetic backgrounds of different fitness (i.e. genetic draft)^4^. Due to the combined effects of genetic drift and variation-reducting selection, the distribution of genetic variation among populations may be uneven and the genetic diversity may be lower than expected predicted by census population size^5,6^. Such decline in genetic diversity may in turn limit the adaptability of populations to environmental change. Gene rescue by gene flow can reduce the threat of local extinction^7^, although the influx of genes may be still constrained by environmental selection^8^.

The population structure of a species distributed across an environmental gradient may be affected by both geographic and environmental factors simultaneously. Autocorrelations of geographic and environmental distances can be additive for genetic differentiation among populations but often confound each other^3,9,10^. The additive effect of such autocorrelations is due not only to the proportional alignment of environmental differences with geographic distance but also to the promotion of divergence by ecological barriers to gene flow^1,3^. That is, when environmental conditions differ, the reduced establishment success of immigrants may accelerate the genetic fixation rate due to the decreased chance of outcrossing, thereby enhancing genetic isolation. This phenomenon not only appears in adaptive loci but could also extend to the whole genome via genetic draft caused by selective sweeps^1^. In this situation, the synergistic effects of environmental-driven and draft selection will lead to positive correlations of both neutral and adaptive loci (instead of adaptive loci only) with environmental differences^3^.

The impact of geographic distance or environmental difference on genetic differences among populations of a species reveals differences in resilience in adapting to heterogeneous environments^11^. Spatial resilience focuses on the patterns and processes of connectivity among locations. The local system resilience may be affected by the geographic distance and environmental heterogeneity among localities. Populations with isolation-by-distance (IBD) reveal positive correlations between genetic distances and geographic distances among populations, in which the genetic diversity turnover relies on the genetic rescue from neighboring populations. By contrast, isolation-by-environment (IBE) indicates populations harboring different genotypes. Variations in genetic composition of IBE populations are sensitive to environmental changes. Populations that are already adapted to alternative environments could harbor higher resilience and potential to adapt to environmental change. Therefore, the resilience of IBE populations is determined by the degree of environmental differences and the adaptability of the population. In addition, terrain and environmental variation may impede the direction and success of dispersal, i.e. isolation-by-resistance (IBR)^12,13^. IBR represents the ecological process or physiological limitation of organisms to dispersal^14^. Lower terrain and environmental resistance and unimpeded habitat connectivity enhance the resilience for species persistence. Since populations affected by IBE potentially have more restricted niche tolerance than a population of a comparable species with only IBD, the mechanism structuring populations must be considered when formulating conservation policies.

An understanding of local adaptation and dispersal limitation can support the development of more appropriate management strategies. For example, when evaluating “single-large or several-small” (SLOSS) strategies for planning a protected area, the several-small strategy and/or collection of germplasm resources from different populations for *ex situ* conservation should be adopted for species with a signature of local adaptation, whereas a single-large strategy may be appropriate for species with IBD or low genetic structure^15^. In other words, the test of IBD, IBE, and IBR can help to understand the process of population genetic differentiation, which will provide a reference for habitat conservation and management of endangered species.

Each plant occupies its own niche, and spatial and resource competition and environmental adaptation determine plants’ distributions^16^. External changes on the landscape and environment in combination with adaptability can also affect their population structure^2,17^, especially in desert areas where the environment is poor. The xeric plant *Ammopiptanthus mongolicus* (Maxim. ex Kom.) Cheng f. (Leguminosae) is the only broad-leaved evergreen shrub in the deserts of eastern Central Asia. *A. mongolicus* is listed as a second-grade vulnerable (VU) plant in the Red List of Threatened Species of China (the Red Book)^18^. Understanding the population genetic structure not only increases the understanding of its demographic dynamics, but also provides information for conservation (e.g., determining management units)^2,19^. Previous research using inter-simple sequence repeat (ISSR) markers concluded that the genetic differentiation of *A. mongolicus* was related to geographic distance, i.e. IBD^20^. Codominant marker evidence (isozymes) indicated that this species is an outcrossed but self-compatible entomophilous plant^21^. The small pollination range of insects and the gravity propagation of seeds were suggested as limiting factors for long-distance gene flow in *A. mongolicus*^20^. *Ammopiptanthus mongolicus* grows in rocky, gravelly, sandy soils of dry valleys, basins, and rocky dunes with a soil depth of less than 30 cm^22^. Most of the desert psammophytes exhibit a spatial distribution strongly associated with scattered fertile soils (i.e., fertile island hypothesis)^23,24^. In contrast, *A. mongolicus* can grow in heterogenous microhabitats^25^. Despite the environmental versatility of *A. mongolicus* at a fine spatial scale, recent studies have shown its global distribution is limited by local climatic conditions (e.g., temperature and humidity), soil organic matter and total nitrogen^26^. Given that environmental variation can critically influence distribution and population sizes of *A. mongolicus*, the isolation-by environment is expected to exert a relevant influence on the genetic structuring of the species. However, previous genetic studies have failed to consider the impact of the local environment on the genetic differentiation of *A. mongolicus* populations^20,27^.

In this study, we explored the effects of geographic distance and local climate on population structure by performing population genetic analyses. We used a multilocus marker, the ISSR, to verify whether *A. mongolicus* populations are genetically differentiated as inferred by Ge *et al*.^20^. We further explored the factors that hinder gene flow among genetically differentiated populations. In addition, since this species has a wide-ranging latitudinal distribution with a varying altitudinal distribution (ranging from ~1000 to 2000 m above sea level), we hypothesized that geographic distance, differences in local climate, and resistance of gene flow to altitudinal and climatic differences drive population genetic differentiation, i.e. isolation-by-distance (IBD), isolation-by-environment (IBE), and isolation-by-resistance (IBR). Accordingly, by outlining the genetic structure and identifying the influencing factors of *A. mongolicus*, conservation suggestions for this endangered Tertiary relict are provided.

## Results

### Low intra-population genetic variation and high inter-population differentiation

**Figure 1:**
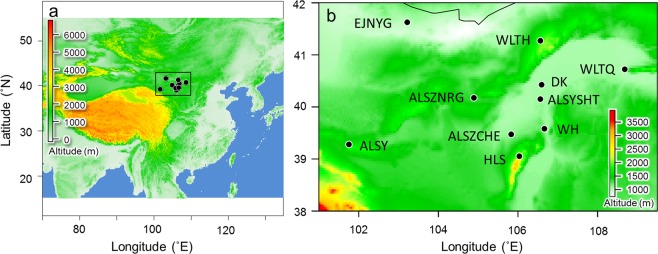
Sampling sites. (**a**) The map shows the relative locations of the distribution of *A. mongolicus* in the inland of temperate Asia; (**b**) the detailed sampling sites in this study and the topographic variation of the distribution. The current altitude layer is publicly available from WorldClim version 2.0^67^ (www.worldclim.org), and the map was generated with the package raster^72^ (http://www.rspatial.org/) in R^58^.

From a total of 200 samples collected from 10 populations (Fig. 1 and Table 1), 105 sharp and clear bands (loci) of the ISSR marker were recorded, of which 71 loci were polymorphic. The genetic diversity estimated from these 71 among-population polymorphic loci revealed that the percentage of within-population polymorphic loci (%P) ranged from 9.86 (EJNYG) to 29.58% (ALSY and WH). The overall expected heterozygosity (*H*_*E*_) was below 0.12 and the Shannon index (*I*) was below 0.16 in all studied populations, with two distant populations (WLTH and ALSY) exhibiting the largest values (Table 2). Although the genetic diversity was slightly higher in the whole species than within populations, it was still low, especially *H*_*E*_ (*I* = 0.452 ± 0.024, *H*_*E*_ = 0.296 ± 0.019, total populations). The high genetic diversity of the total population relative to each single population suggests high differentiation among the populations.

**Table 1:** Geographic coordinates of the sampling sites.

Population code	Population source	Longitude (°E)	Latitude (°N)	Altitude (m)
ALSZNRG	Alashan Zuo Banner NUOERGONG	104°53′45″	40°10′30″	1412
EJNYG	Ejina Yagan	103°13′12″	41°37′37″	1002
ALSYSHT	Alashan You Banner Suhaitu	106°33′42″	40°09′10″	1059
HLS	Helanshan	106°01′57″	39°03′14″	2048
DK	Bayannur Dengkou	106°35′43″	40°25′43″	1056
ALSZCHE	Alashan Zuo Banner Chahaer	105°49′49″	39°28′25″	1146
WLTH	Wulate Hou Banner	106°33′42″	41°16′29″	1822
ALSY	Alashan You Banner	101°45′17″	39°16′50″	1595
WH	Wuhai	106°39′49″	39°34′54″	1114
WLTQ	Wulate Qian Banner	108°40′57″	40°43′28″	1184

**Table 2:** Population genetic diversity of *A. mongolicus* estimated by 71 polymorphic ISSR loci.

Pop	*N*	%*P*	*N*_*A*_	*N*_*E*_	*I*	*H*_*E*_	*UH*_*E*_
ALSZNRG	20	14.08	0.775 ± 0.081	1.061 ± 0.023	0.061 ± 0.019	0.038 ± 0.013	0.040 ± 0.014
EJNYG	20	9.86	0.690 ± 0.077	1.052 ± 0.021	0.049 ± 0.019	0.032 ± 0.012	0.034 ± 0.013
ALSYSHT	20	23.94	0.986 ± 0.084	1.131 ± 0.034	0.117 ± 0.027	0.077 ± 0.019	0.081 ± 0.020
HLS	20	19.72	0.887 ± 0.084	1.093 ± 0.028	0.088 ± 0.023	0.057 ± 0.016	0.060 ± 0.017
DK	20	25.35	1.028 ± 0.083	1.134 ± 0.032	0.124 ± 0.027	0.081 ± 0.019	0.086 ± 0.020
ALSZCHE	20	22.54	0.958 ± 0.084	1.111 ± 0.030	0.105 ± 0.025	0.068 ± 0.017	0.072 ± 0.018
WLTH	20	28.17	1.225 ± 0.064	1.184 ± 0.040	0.154 ± 0.031	0.105 ± 0.022	0.110 ± 0.023
ALSY	20	29.58	1.127 ± 0.080	1.193 ± 0.042	0.158 ± 0.032	0.107 ± 0.022	0.113 ± 0.023
WH	20	29.58	1.056 ± 0.087	1.154 ± 0.036	0.139 ± 0.029	0.091 ± 0.020	0.096 ± 0.021
WLTQ	20	16.90	0.817 ± 0.083	1.093 ± 0.028	0.085 ± 0.024	0.056 ± 0.016	0.059 ± 0.017
Total	200	100.00	2.000 ± 0.000	1.491 ± 0.039	0.452 ± 0.024	0.296 ± 0.019	0.297 ± 0.019

*N*, sample size; %*P*, percentage of polymorphic loci; *N*_*A*_, number of different alleles; *N*_*E*_, number of effective alleles; *I*, Shannon’s information index; *H*_*E*_, expected heterozygosity; *UH*_*E*_, unbiased *H*_*E*_ estimated by the equation *H*_*E*_ *×* *N*/(*N* − 1).

**Figure 2:**
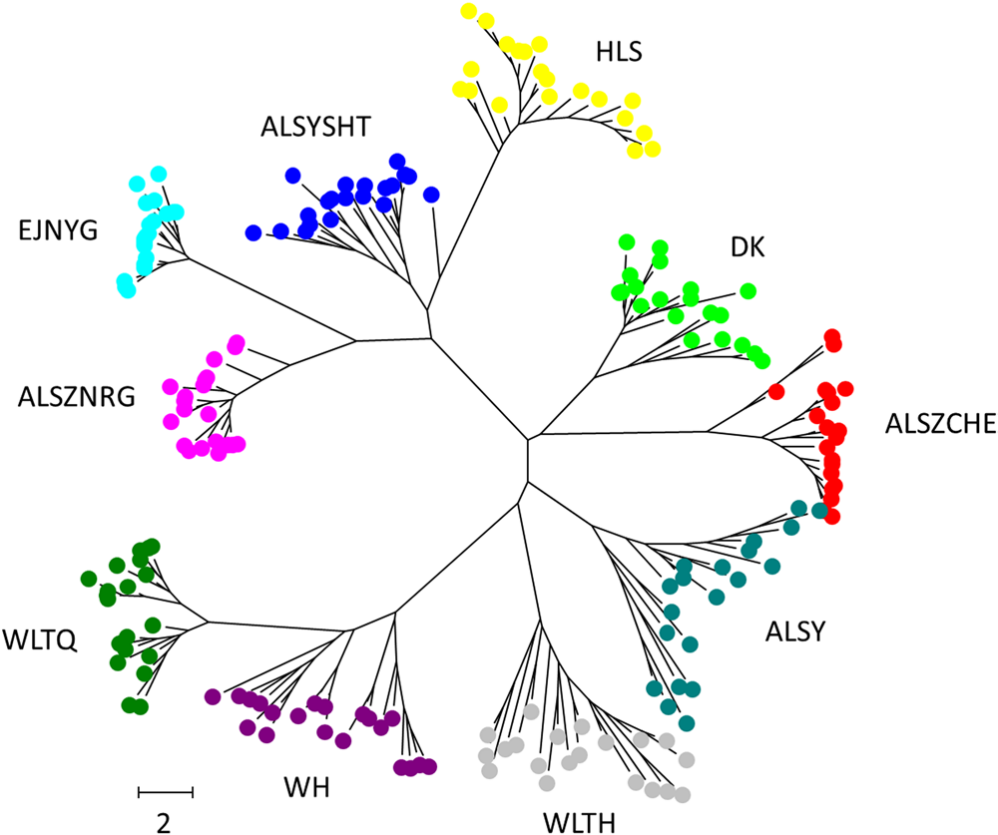
Unrooted neighbor-joining tree showing the clear genetic clusters among populations.

**Figure 3:**
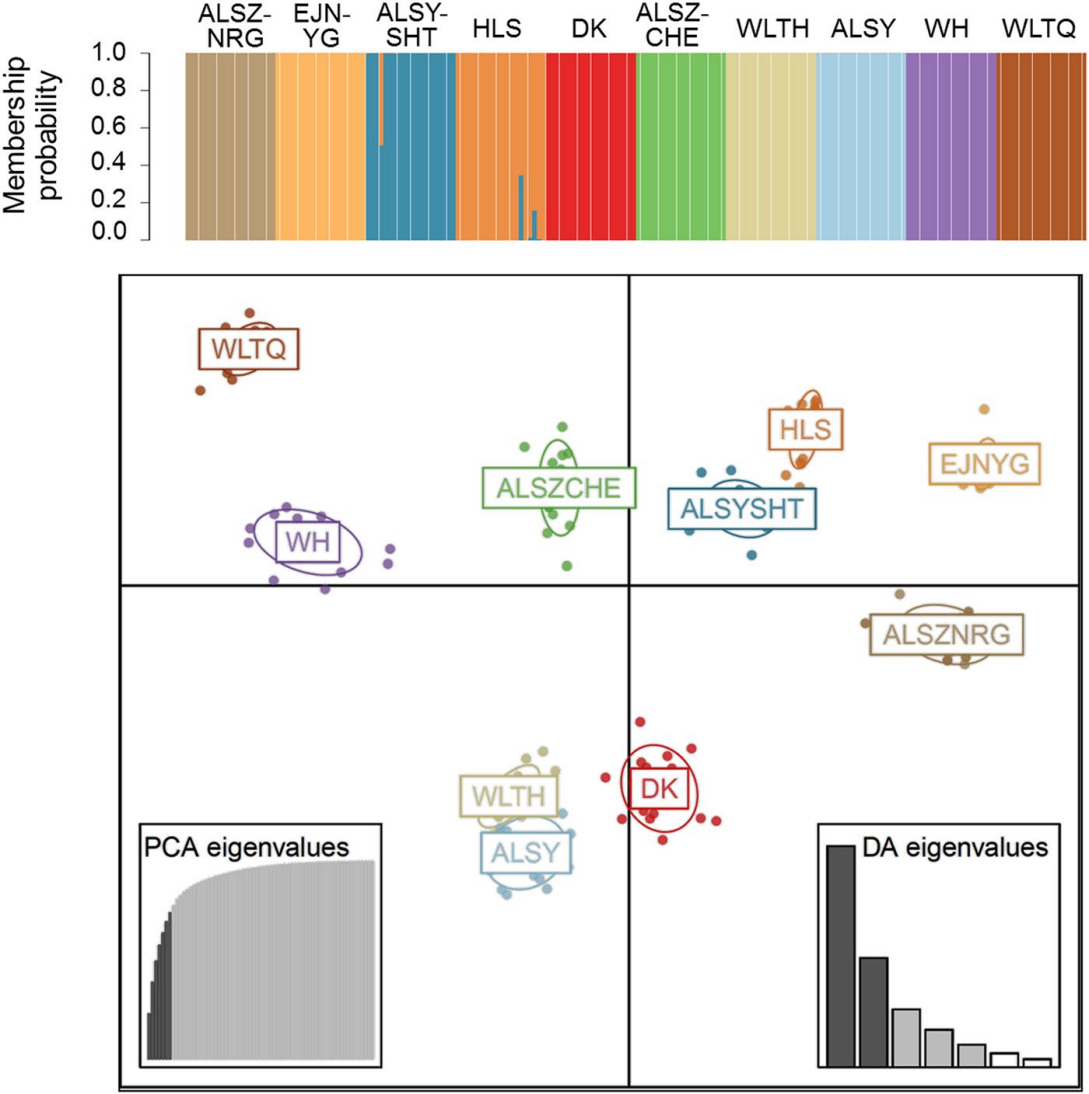
The results of discriminant analysis of principal components (DAPC). The upper and lower plots are the component plot and scatterplot, respectively. The DAPC was conducted based on seven PCs and five discriminant functions that conserved 73.8% of the genetic variation.

AMOVA was used to assess the population genetic structure and revealed that 76.58% of the genetic variation was partitioned among populations, while the remaining 23.42% was attributed to differences between individuals within populations (Table 3). The inference of high genetic differentiation was also confirmed by both the neighbor-joining (NJ) tree (Fig. 2) and discriminant analysis of principal components (DAPC, Fig. 3). Our results supported the existence of ten genetic clusters, indicating that each population had its own genetic signature. In DAPC, seven principal components (PCs) were retained according to the 1000-run K-means algorithm assessed by the Bayesian information criterion (BIC), and the optimal number of clusters was 10, which corresponds to the number of sampled populations. Figure 3 shows both the component and scatter plots; all samples were clearly assigned to their own populations, except one sample in population ALSYSHT with roughly half of its genetic component from HLS and a few samples of HLS with small proportions of genetic admixture with ALSYSHT (the component plot of Fig. 3).

**Table 3:** Analysis of molecular variance (AMOVA) of 10 populations of *A. mongolicus*.

Source	df	SS	MS	Est. Var.	%	*Φ*_ST_	P
Among pops	9	1592.335	176.926	8.713	76.58%	0.766	0.010
Within pops	190	506.250	2.664	2.664	23.42%		
Total	199	2098.585		11.378	100.00%

**Figure 4:**
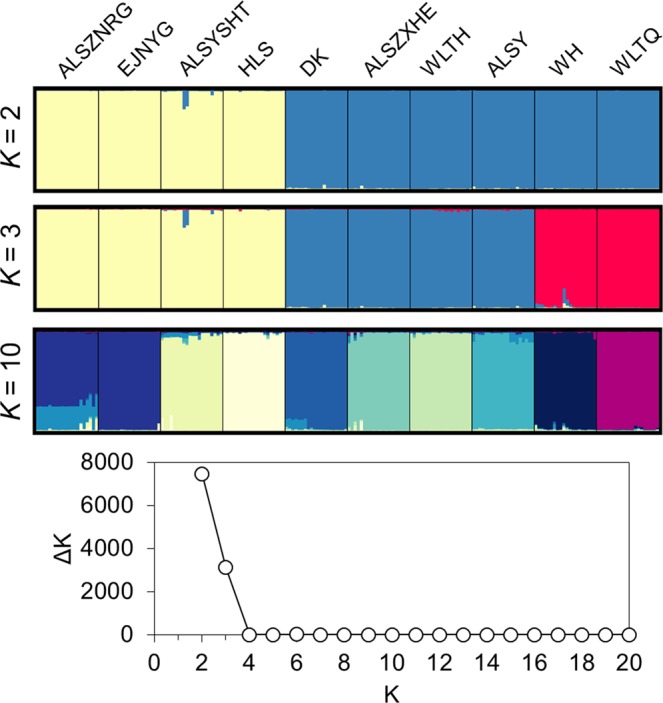
Patterns of genetic clustering inferred by Bayesian clustering analysis (STRUCTURE). The optimal and second best grouping number (*K* = 2 and 3) were inferred by the Δ*K* (the bottom graph). *K* = 10 is shown to present the genetic admixture pattern among populations.

STRUCTURE analysis showed that the optimal grouping number (*K*) of genetic components was two based on the logarithmic probability change rate of successive *K*-value data. When *K* = 2, the populations ALSZNRG, EJNYG, ALYSHT, and HLS clustered together, while the remaining populations formed another group (Fig. 4). Except for two ALYSHT samples, only weak genetic admixture was detected between these two groups (Table 3). The second best *K* was three, with the WH and WLT populations forming the third group (Fig. 8). The grouping pattern of STRUCTURE was consistent with that of the NJ tree (Fig. 2). When *K* = 10, almost every population had its own unique genetic components, except for a composite component in ALZBURG similar to a part of EJNYG (Fig. 4). Although there were slight differences, the STRUCTURE, DAPC and NJ tree analyses yielded congruent inferences of obvious genetic differentiation.

### IBE explains the population structure of *A. mongolicus*

**Figure 5:**
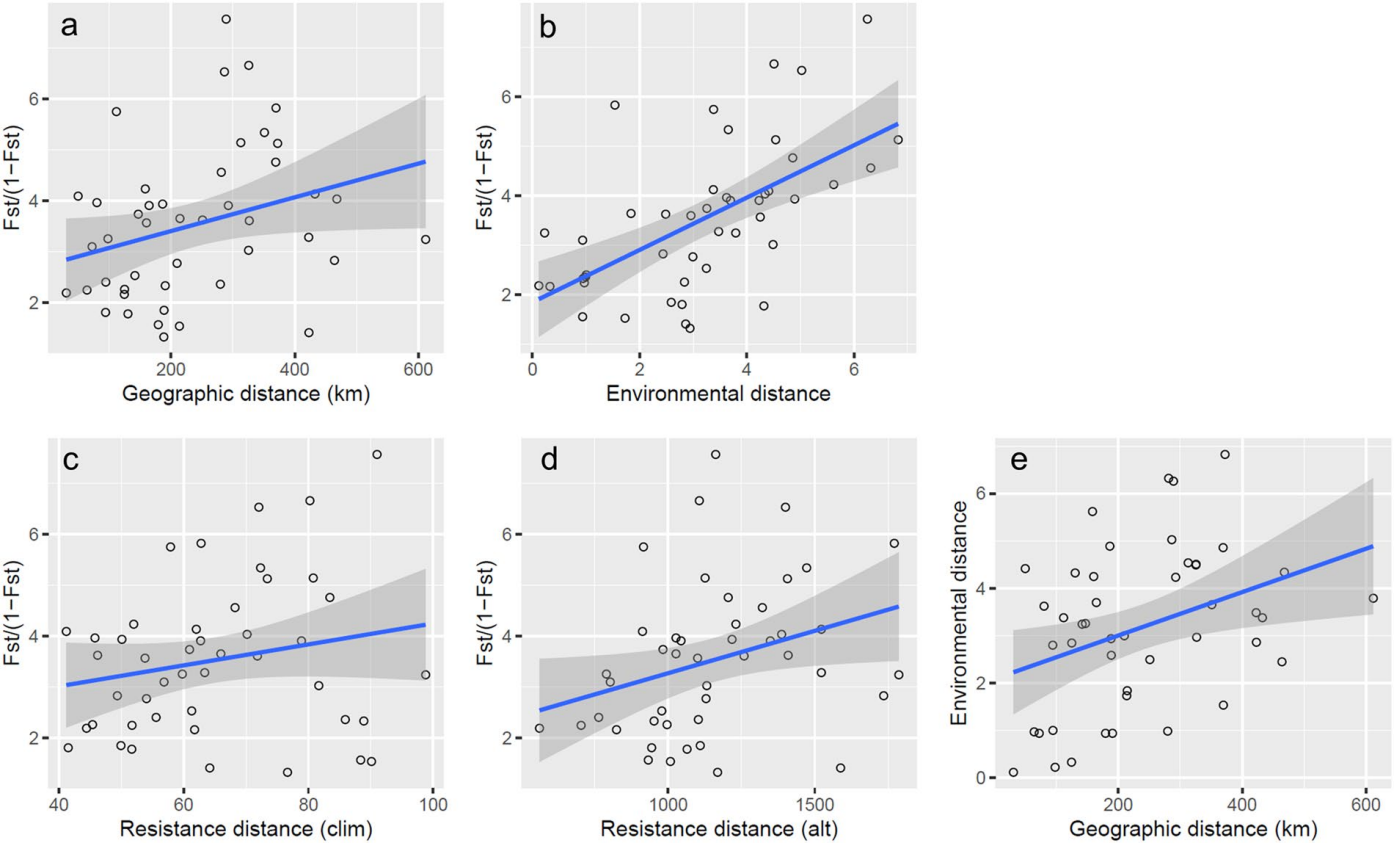
Linear regression lines showing the correlations among genetic, geographic, and environmental distances. (**a**) The test of isolation-by-distance (IBD); (**b**) the test of isolation-by-environment (IBE); (**b**) the test of isolation-by-resistance in climate (IBR_clim_); (**d**) the test of isolation-by-resistance in altitude (IBR_alt_); (**e**) the test of correlation between geographic and environmental distance. Among these linear relationships, only the climatic distance was significantly correlated with genetic distance (i.e. IBE), as supported by the Mantel test, partial Mantel test, and MLPE (Table 4).

Models of IBD, IBE, and IBR were tested to explain the genetic differentiation patterns among populations of *A. mogolicus*. The results of the partial Mantel test suggested that the population genetic structure could be explained by IBE (*r* = 0.609, *p* = 0.001) instead of IBD (*r* = 0.137, *p* = 0.212), IBR_clim_ (*r* = −0.031, *p* = 0.254), or IBR_alt_ (*r* = 0.179, *p* = 0.250) (Table 4 and Fig. 5). However, there was a marginally significant correlation between geographic distance and environmental difference (Mantel test, *r* = 0.368, *p* = 0.073), implying that the farther the geographic distance, the greater the environmental difference. Model selections for the maximum likelihood population effect mixed effect (MLPE) revealed that the IBE was the first-ranked model explaining the population genetic structure according to the ranking of the AIC and BIC (Tables 4 and 5), consistent with the inference of the partial Mantel test. The second ranked model was IBR_clim_, with only a small difference in AIC compared to the IBE model (ΔAIC = 0.25, Tables 4 and 5). Both IBE and IBR_clim_ attribute population genetic structure to the climatic effect; the former explains the impact of climate differences on the survival and reproduction of colonizers, while the latter emphasizes the facilitation or inhibition of the migration (gene flow) process of organisms by climate differences. However, despite small ΔAIC between IBE and IBR_clim_, the effect size of the fixed effect (climatic composite resistance distance) is small (fixed estimate = 0.020) in IBR_clim_, suggesting that the environmental resistance during migration contributes less than the selective pressure after colonization.

**Table 4:** Summary results of the Mantel test, partial Mantel test, and the model selection for the maximum likelihood population effect mixed model (MLPE).

Model	Mantel		partial Mantel		MLPE									
	*r*	*P*	*r*	*P*	Fixed estimate	Fixed SE	Random estimate	Random SE	df	AIC	ΔAIC	BIC	logLik	deviance
**IBE**	**0.449**	**0.020**	**0.609**	**0.001**	**0.155**	**0.119**	**0.748**	**0.865**	**4**	**146.76**	**0**	**153.99**	**−69.382**	**138.76**
IBR_clim_	0.179	0.231	−0.031	0.254	0.020	0.016	0.850	0.922	4	147.01	0.25	154.23	−69.503	139.01
IBD	0.338	0.084	0.137	0.212	−0.001	0.002	1.004	1.002	4	148.34	1.58	155.56	−70.168	140.34
IBR_alt_	0.378	0.075	0.179	0.250	−0.0003	0.001	0.830	0.911	4	148.44	1.68	155.66	−70.219	140.44

In the MLPE test, only four single fixed factor models are listed. The results of model selection are shown in Table 5. The order of the models corresponds to their ranking from best (smallest AIC and BIC) to worst. The first-ranked model is marked in bold.

**Table 5:** Model selection for 15 MLPE models.

Models	df	AIC	ΔAIC	BIC	logLik	deviance
**D**_**gen**_ **~ D**_**env**_	**4**	**146.76**	**0**	**153.99**	**−69.382**	**138.76**
D_gen_ ~ R_clim_	4	147.01	0.25	154.23	−69.503	139.01
D_gen_ ~ D_env_ + R_clim_	5	147.57	0.56	156.61	−68.788	137.57
D_gen_ ~ D_geo_	4	148.34	0.77	155.56	−70.168	140.34
D_gen_ ~ D_geo_ + D_env_	5	148.39	0.05	157.42	−69.194	138.39
D_gen_ ~ R_alt_	4	148.44	0.05	155.66	−70.219	140.44
D_gen_ ~ D_geo_ + R_clim_	5	148.48	0.04	157.52	−69.242	138.48
D_gen_ ~ D_env_ + R_alt_	5	148.50	0.02	157.54	−69.252	138.50
D_gen_ ~ R_clim_ + R_alt_	5	148.76	0.26	157.79	−69.380	138.76
D_gen_ ~ D_geo_ + D_env_ + R_clim_	6	148.81	0.05	159.65	−68.406	136.81
D_gen_ ~ D_env_ + R_clim_ + R_alt_	6	149.12	0.31	159.96	−68.562	137.12
D_gen_ ~ D_geo_ + R_alt_	5	150.30	1.18	159.33	−70.149	140.30
D_gen_ ~ D_geo_ + R_clim_ + R_alt_	6	150.34	0.04	161.18	−69.168	138.34
D_gen_ ~ D_geo_ + D_env_ + R_alt_	6	150.38	0.04	161.22	−69.191	138.38
D_gen_ ~ D_geo_ + D_env_ + R_clim_ + R_alt_	7	150.74	0.36	163.38	−68.368	136.74

The order of the models corresponds to their ranking from best (smallest AIC and BIC) to worst. The first-ranked model is marked in bold.

D_gen_, D_geo_, D_env_, R_clim_, and R_alt_ denote the genetic distance (i.e. *F*_ST_/(1 − *F*_ST_)), geographic distance, environmental difference, and resistance distances estimated from climatic composite resistance surface and from altitudinal resistance surface among populations, respectively.

**Figure 6:**
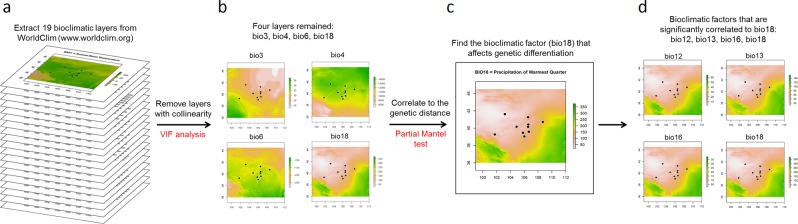
Flow chart of the experimental design to further filter the environmental factors affecting population genetic structure. (**a**) Bioclimatic layers were extracted from the open database WorldClim version 2.0^67^ (www.worldclim.org); (**b**) factors (layers) with multicollinearity were removed using variance inflation factor (VIF) analysis, and factors with VIF > 10 were discarded; (**c**) the remaining bioclimatic factors (bio3, bio4, bio6, and bio18) were correlated with the genetic distance by the partial Mantel test, and the key bioclimatic factor (bio18) was identified; (**d**) factors related to the key bioclimatic factor were identified (bio12, bio13, bio16, and bio18).

**Figure 7:**
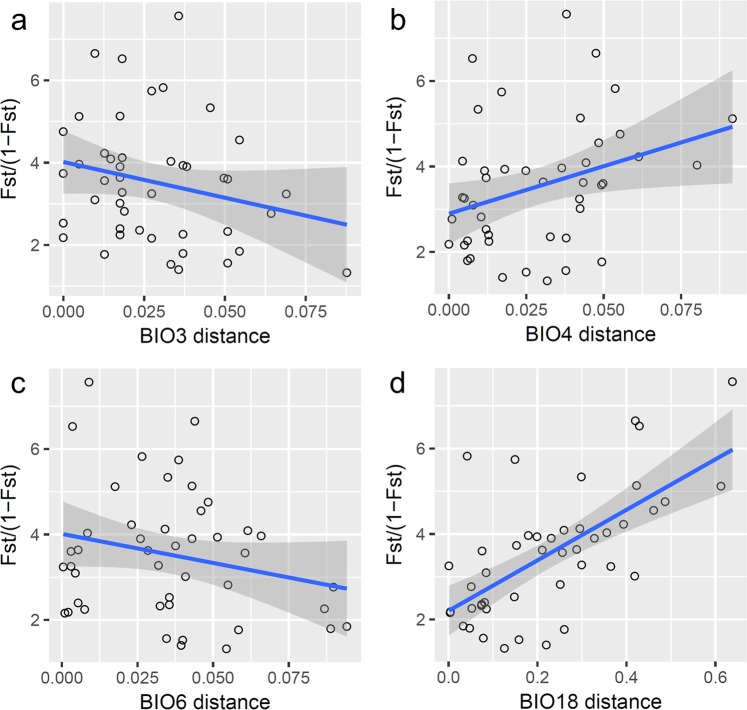
Plots of linear regressions showing the correlations between genetic distance and differences in each single bioclimatic factor. (**a**) Genetic distance vs. bio3 (partial Mantel test: *r* = −0.361, *p* = 0.934, conditioning on geographic distance); (**b**) genetic distance vs. bio4 (partial Mantel test: *r* = 0.287, *p* = 0.127, conditioning on geographic distance); (**c**) genetic distance vs. bio6 (partial Mantel test: *r* = −0.095, *p* = 0.645, conditioning on geographic distance); (**d**) genetic distance vs. bio18 (partial Mantel test: *r* = 0.553, *p* = 0.007, conditioning on geographic distance).

**Figure 8:**
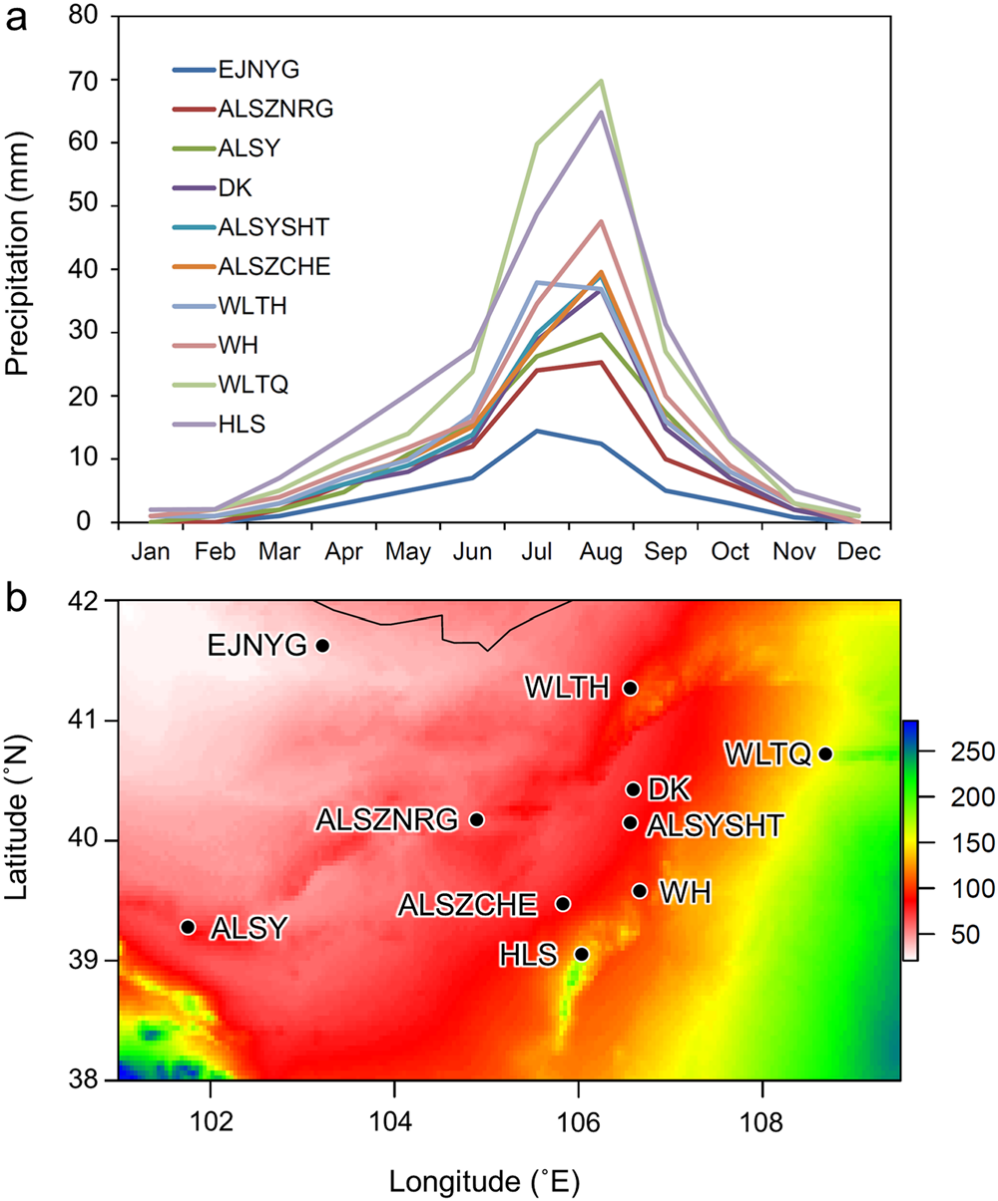
Differences in precipitation among the *A. mongolicus* sampling sites. (**a**) Monthly precipitation of the sampled populations; (**b**) graphic layer of bio18 showing the gradient of precipitation of the warmest quarter among the sampling sites.

The Mantel test has been criticized for high Type 1 error due to multicollinearity^9,28^. Therefore, we removed the bioclimatic factors with multicollinearity and conducted the partial Mantel test again using each retained single factor (Fig. 6) to further explore which bioclimatic variable is the key factor affecting population genetic differentiation. Four bioclimatic factors, bio3, bio4, bio6, and bio18, were retained; only bio18 (precipitation of the warmest quarter) was positively correlated with the genetic distance (*r* = 0.553, *p* = 0.007, Fig. 7). Since some bioclimatic factors were removed due to collinearity with bio18, we tested environmental distances based on these individual factors (bio12, bio13, and bio16) for correlations with the genetic distance among populations, which confirmed their positive correlations with genetic distance (bio12: *r* = 0.563, *p* = 0.005; bio13: *r* = 0.554, *p* = 0.003; bio16: *r* = 0.567, *p* = 0.004, by partial Mantel test, conditioning on geographic distance). Bio12 (annual precipitation), bio13 (precipitation of the wettest month), bio16 (precipitation of the wettest quarter), and bio18 (precipitation of the warmest quarter) are all bioclimatic dimensions related to precipitation. According to the monthly precipitation records (Fig. 8a), the annual precipitation mostly accumulates from June to September. The regional precipitation not only decreases westward but is also obviously related to topography (Fig. 8b).

Univariate MLPE regression was also conducted to test the IBE model with each of the 19 bioclimate distances as the fixed variable and the population effect as the random variable. The ranked AIC revealed that both models with bio17 and bio19 as the fixed variable had the smallest AIC values and significantly better fits than the other models (ΔAIC > 5, Table 6). Bio17 and bio19 are the precipitation of the driest and coldest quarters, respectively. In our study area, the coldest and driest seasons are the same, resulting in the same estimates in bio17- and bio19-univariate MLPE. Although the most crucial climatic factor affecting the genetic distance differed between the partial Mantel test (summer precipitation) and MLPE (winter precipitation), both analyses suggest that the regional precipitation difference is the key factor affecting the genetic structure of *A. mongolicus*.

**Table 6:** Summary results of the MLPE and the model selection.

Fixed factor	Fixed estimate	Fixed SE	Random estimate	Random SE	Df	AIC	ΔAIC	BIC	logLik	deviance
**bio17**	**1.475**	**0.435**	**0.498**	**0.705**	**4**	**138.51**	**0.00**	**145.74**	**−65.256**	**130.51**
**bio19**	**1.475**	**0.435**	**0.498**	**0.705**	**4**	**138.51**	**0.00**	**145.74**	**−65.256**	**130.51**
bio4	26.825	9.793	0.356	0.597	4	143.52	5.01	150.75	**−**67.762	135.52
bio7	28.168	13.450	0.503	0.709	4	144.89	6.38	152.12	**−**68.445	136.89
bio2	**−**11.449	6.842	0.877	0.937	4	146.00	7.49	153.22	**−**68.997	138.00
bio8	6.699	4.468	0.588	0.767	4	146.57	8.06	153.80	**−**69.285	138.57
bio1	**−**1.451	1.529	0.761	0.872	4	147.65	9.14	154.87	**−**69.824	139.65
bio14	**−**0.315	0.360	0.730	0.854	4	147.78	9.27	155.01	**−**69.890	139.78
bio9	**−**2.687	3.068	0.718	0.847	4	147.79	9.28	155.01	**−**69.894	139.79
bio6	**−**4.650	6.977	0.727	0.853	4	148.10	9.59	155.33	**−**70.052	140.10
bio12	0.965	1.400	0.669	0.818	4	148.14	9.63	155.37	**−**70.069	140.14
bio3	**−**5.184	10.973	0.767	0.876	4	148.31	9.80	155.54	**−**70.157	140.31
bio16	0.736	1.426	0.695	0.833	4	148.31	9.80	155.54	**−**70.155	140.31
bio13	0.661	1.312	0.694	0.833	4	148.32	9.81	155.55	**−**70.162	140.32
bio11	**−**1.257	3.052	0.741	0.861	4	148.37	9.86	155.60	**−**70.186	140.37
bio18	0.624	1.452	0.706	0.840	4	148.38	9.87	155.61	**−**70.191	140.38
bio15	**−**1.907	6.145	0.768	0.876	4	148.44	9.93	155.67	**−**70.221	140.44
bio10	**−**0.856	4.193	0.777	0.882	4	148.50	9.99	155.72	**−**70.248	140.50
bio5	**−**0.920	5.278	0.777	0.882	4	148.51	10.00	155.74	**−**70.254	140.51

The order of the models corresponds to their ranking from best (smallest AIC and BIC) to worst. The first-ranked models are marked in bold.

## Discussion

### IBE is the best model on population structure

The population genetic structure of *A. mongolicus* was previously suggested to fit the IBD model^20^, which implies an inverse proportion of effective dispersal to geographical distance^29–31^. Over the past decade, accumulating studies have indicated that geographic distance or geographic barriers may not be the only factor affecting gene flow. Environmental differences may be the key factor underlying effective migration^9,10,31^. In this study, we suggest that the adaptability of *A. mongolicus* to local climate affects its seed germination and colonization. The effect of selection pressure on population differentiation is usually faster than that of drift and could occur at a small geographic scale^32–34^. Notably, environmental differences were marginally correlated with geographic distance. We therefore suggest that the previous inference of IBD^20^ could be due to the intercorrelation between geographic and environmental differences. The increasing number of open databases is now helping to clarify ecological and evolutionary phenomena. A meta-analysis showed that 74.3% of phylogeographic studies (52 of 70 studies) revealed significant IBE patterns, including 37.1% (27 studies) revealing spatial autocorrelation (i.e. covariates with IBD)^9^. Similarly, from 106 IBE studies, Shafer and Wolf^10^ reported effect sizes of 0.34 (95% CI 0.24–0.42) and 0.26 (95% CI 0.13–0.37) for a mixed-effect model with and without controlling spatial autocorrelation, respectively, suggesting that spatial autocorrelation reduces IBE correlations for environmental variables. These studies indicated the relevance of environmental autocorrelation for the spatial effect (i.e. IBD). That is, the previous inference that the population differentiation of *A. mongolicus* aligns with geographic distance^20^ probably reflects differential adaptation to the local climate. Differential adaptability to heterogeneous environments provides a better explanation than IBD in *A. mongolicus*, i.e. divergent selection is more important than neutral processes.

### Genetic draft explains low genetic diversity

The low estimates of genetic diversity are consistent with the previous estimation by Ge *et al*.^20^, which included populations located farther south but no populations in Alashan (ALSY, ALSZNRG, ALSYSHT, and ALSZCHE). The genetic diversity of *A. mongolicus* was also lower than that of other desert species estimated by ISSR, e.g. *Achillea fragrantissima* in Egypt^35^, *Citrullus colocynthis* in India^36^, and *Lasiurus sindicus* in India^37^. Although the factors affecting the genetic diversity of species vary, the selection pressure of local precipitation with genetic draft may be the limiting factor affecting the genetic variation of desert plant populations, such as *A. mongolicus* in this case.

Environmental heterogeneity would reduce the chance of dispersal^38,39^, and the constraint of the range of distribution may lead to deleterious erosion of genetic diversity due to increased inbreeding and genetic draft^39^. Precipitation is an important limiting factor for the reproductive success of desert plants. The growth pattern of *A. mongolicus* is similar to that of desert deciduous plants or summer annuals, with blossoming and germination during the high-rainfall season^40^. Rapid blooming is advantageous for plant reproductive success in the desert^40^. However, pollinators tend to visit flowers of the same or adjacent plants instead of distant flowers in the short blooming season, which may reduce the outcrossing rate of *A. mongolicus* (inbreeding coefficient *F*_IS_ > 0 in all loci^21^).

In addition, rainfall restrictions in deserts may also result in strong selection on *A. mongolicus*. With a selective sweep, genetic variation of adjacent genes decreases along with adaptive loci, which will even expands to most genome regions. Compared with other plants that may also be affected by genetic draft, such as *Dactylis glomerata* L. in the plateau of Central Asia and Western China^41^, *A. mongolicus* exhibits extremely low intra-population genetic variation, suggesting that regional environmental pressures (especially precipitation) in the desert have a more severe impact on this broad-leaved green plant. Rainfall-induced declines in outcrossing opportunities and strong selective sweeps could explain the low genetic variation of *A. mongolicus*, which may also be resistant to the rescue effect of gene flow among populations.

### Differential local precipitation is the key to population differentiation

Summer rainfall almost completely determines the annual precipitation in the distribution of *A. mongolicus*. In general, the annual precipitation tends to decline in a southeast-to-northwest direction across the Asian continent^42^, but fluctuations in terrain (e.g. the Hetao Plain, Helan Mountains, and Mongolian Highlands) make the local climate more complicated. Such local differential precipitation may have long been the selective pressure not only for the breeding and dispersion of *A. mongolicus* but also for the water supply in the dry season.

Several studies have indicated that water is the key factor affecting the seed germination^43^ and seedling growth^44^ of *A. mongolicus*. In summer (July and August), the legume of *A. mongolicus* is ripe and dehiscent, and seeds fall off, quickly absorb water and germinate^43^. In a manipulation experiment, 85% of seedlings wilted in a 5-day drought treatment^44^, indicating that the demand for water is a limiting factor for the regeneration of *A. mongolicus*. Due to the lack of defoliation in winter, supplementing evapotranspiration with some precipitation may also affect *A. mongolicus* survival in winter. Although the local precipitation is small and varies little in winter (the cumulative precipitation ranges from 0 to 6.06 mm in Dec~Feb), such differences may cause local adaptation. Differential adaptability to precipitation among populations might accelerate the process of population differentiation by stalling maladaptive immigration.

As described above, differential local precipitation might also affect pollinators’ species and visiting frequency^45^. Differences in precipitation could vary the ratio of bee pollinators to fly pollinators; the former require dry soil for nesting, whereas the latter require moist environments for larval growth and metamorphosis^46^. Changes in the abundance of pollinators could affect the success of pollination and seed yield, even though the connectivity of the plant and pollinator relationship may not be disturbed by precipitation^47^. In addition, precipitation can also affect rhizobium symbionts^48^ and pathogen infectivity^49^, thereby affecting plant health and population regeneration. The presence of some endosymbiotic fungi (dark septate endophytes) can facilitate the growth of *A. mongolicus* under drought conditions^50^. We have not explored differences in soil microorganisms and endophytes among different populations, but distributional differences of these endophytes may also cause differential adaptability to precipitation among populations. The local adaptation caused by differential precipitation may lead to divergent directions of genetic draft, which may explain apparent population differentiation in *A. mongolicus*.

### Concluding remarks

In conclusion, the selective pressure of the environmental gradient (differences in precipitation) is strong for *A. mongolicus* and likely explains the low genetic variation within populations and high population differentiation. Most individuals carry not only locally adapted genes but also homogenized genomic variation, which decreases successful emigration to populations with different environments, i.e. selection against maladapted dispersers^12,31^. *A. mongolicus* is the only evergreen shrub in the desert of Northwest China and is an important wintering place for several small animals, i.e. an umbrella species. Given the low genetic variation within populations and maladapted gene flow among populations, every population is a unique evolutionarily significant unit and should be considered as a unique management unit for conservation. The high dependence of adaptability on precipitation is not propitious for effective gene flow among populations. Therefore, the establishment of ecological corridors^51–53^ may not be an appropriate strategy for conservation. Germplasms from different populations should be actively preserved to maintain the complete gene pool and increase the evolutionary resilience^9^ of *A. mongolicus* in the face of increasingly severe climate change.

## Materials and Methods

### Species studied and sampling

The genus *Ammopiptanthus* was suggested to have originated from the broad-leaved evergreen Tethyan flora^54^, as supported by molecular dating indicating that the genus *Ammopiptanthus* split from its sister taxa in the early Miocene (chloroplast DNA *matK* sequences:19.6 Mya; nuclear ITS sequences: 21.8 Mya)^55^. *Ammopiptanthus mongolicus* is discontinuous distributed in western Inner Mongolia, northern Ningxia and Northern Gansu in China, ranging from 36°27′N–42°01′N, 102°36′E–108° 49′E^26^. The sampling area of this study was the core distribution of *A. mongolicus* in Inner Mongolia, China. We chose 10 populations covering the main distribution range of *A. mongolicus* (Table 1). Fresh leaves were sampled from 20 individuals per population, and each sampled plant was distant from other plants by at least 20 meters. A total of 200 individuals were sampled. The sampled leaves were placed immediately in a liquid nitrogen tank and stored in a −20 °C refrigerator after carrying to the laboratory.

### Molecular techniques

Genomic DNA was extracted using a commercial kit DNAquick Plant System (TIANGEN Biotech Co., Ltd., Beijing, China). DNA quality was checked by agarose gel electrophoresis and by the DNA absorbance ratio (OD_260_/OD_280_: 1.7~1.9) in a WD-9403C UV Viewing Cabinet (BEIJING LIUYI Biotechnology Co., Ltd., Beijing, China). ISSR amplification was performed using fifteen primers (Table 7) with the following PCR procedure: denaturation at 95 °C for 5 min, followed by 35 cycles of denaturation at 95 °C for 1 min, annealing at the proper temperature for 1 min (Table 7), and extension at 72 °C for 1 min, with a final 10-min extension at 72 °C. PCR was conducted in an MJMini personal thermal cycler (Bio-Rad, Hercules, USA) and T100™ thermal cycler (Bio-Rad, Hercules, USA). All PCR products were checked by 1.5% agarose gel electrophoresis, and the appearance of bands was read. Ghost bands were excluded by comparison with a negative control in which water was used as the template with the same ISSR protocol. The ISSR experiments were repeated twice to ensure that the peak signals affirming the bands (loci) were not PCR errors. Only loci that were consistently present or absent in all preliminary tests were read in the formal experiment.

**Table 7:** Bands and reaction conditions of ISSR primers.

Primers	Total bands	Polymorphic bands	Sequence(5′ to 3′)	Annealing temperature (°C)
UBC-808	8	5	AGAGAGAGAGAGAGAGC	42
UBC-809	6	5	AGAGAGAGAGAGAGAGG	42
UBC-811	8	7	GAGAGAGAGAGAGAGAC	42
UBC-813	6	1	CTCTCTCTCTCTCTCTT	40
UBC-834	6	5	AGAGAGAGAGAGAGAGYT	42
UBC-842	6	5	GAGAGAGAGAGAGAGAYG	44
UBC-859	7	5	TGTGTGTGTGTGTGTGRC	44
UBC-876	10	10	GATAGATAGACAGACA	43
UBC-855	7	5	ACACACACACACACACYT	42
UBC-840	7	2	GAGAGAGAGAGAGAGAYT	42
UBC-880	7	4	GGAGAGGAGAGGAGA	50
UBC-881	6	3	GGGTGGGGTGGGGTG	48
UBC-886	7	4	VDVCTCTCTCTCTCTCT	41
UBC-888	8	6	BDBCACACACACACACA	41
UBC-889	6	4	DBDACACACACACACAC	40
Total	105	71		

### Genetic diversity and population genetic structure

The genetic diversity was estimated by the indices of percentage of polymorphic loci (%P), average number of different alleles per locus (*N*_*A*_), effective number of alleles per locus (*N*_*E*_), Shannon’s information index (*I*), expected heterozygosity (*H*_*E*_), and unbiased heterozygosity (*UH*_*E*_) using GenAlEx v. 6.5^56^. The contributions of genetic variation between and within populations were assessed by analysis of molecular variance (AMOVA). The significance of genetic differentiation between populations was estimated by *Φ*_ST_ under 999 permutations. We also conducted DAPC to determine if the spatially structured population was also genetically structured using the package adegenet^57^ in R^58^. The best clustering number (*k*) was inferred by the *k*-means algorithm with 10^6^ simulations evaluated by BIC (the elbow in the BIC curve and the smallest BIC). The optimal number of PCs retained for DAPC was evaluated by a-score optimization. A component plot and scatter plot were drawn to illustrate the population clustering pattern of *A. mongolicus*. Patterns of genetic admixture were assessed by Bayesian clustering analysis, a population model-based approach based on Hardy-Weinberg and linkage equilibria^59^, with the assistance of STRUCTURE 2.3.4^60^. We estimated the posterior probability of the grouping number (*K* = 1–20) by 10 independent runs using 10^6^ steps of Markov chain Monte Carlo (MCMC) replicates after 10% burn-in for each run to evaluate consistency. The best grouping number was evaluated by Δ*K*^61^ in STRUCTURE HARVESTER ver. 0.6.94^62^. In addition, to understand the relationships between each of the lineages, we transformed the number of differences in ISSR loci between individuals into a triangular matrix and then constructed an NJ tree using MEGA6^63^.

### Testing IBD, IBE, and IBR

To test the effects of geographic distance and environmental differences on genetic structure, the partial Mantel test was conducted using the R package vegan^64^. We calculated the pairwise genetic distances among populations using *F*_ST_/(1 − *F*_ST_)^65^. Euclidean distances of geographic distance were calculated using the R package fossil^66^. We also collected 19 standard bioclimatic variables of 10 sampling sites as environmental data from WorldClim version 2.0^67^. We considered the 19 bioclimatic variables as different environmental space vectors and used the Canberra distance to calculate the distance between populations in this vector space. To test IBD, the genetic distance was used as the response, the geographic distance as the predictor, and the environmental distance as the condition factor. To test IBE, the roles of environmental distance and geographic distance were interchanged. In addition, to test whether these environmental factors impeded gene flow, the climatic composite resistance surface was transformed from raster layers of the bioclimatic variables using Circuitscape 4.0^68^. We also transformed the altitudinal layer into an altitudinal resistance surface. These two resistance surfaces were used to test IBR, namely IBR_clim_ and IBR_alt_, respectively. The Mantel statistic was based on Spearman’s rank correlation with 9999 permutations.

Mantel and partial Mantel tests have been strongly criticized for inflated type-I error, potential collinearity between environmental variables when building an environmental dissimilarity matrix, low power, etc.^9,28,69^. Therefore, we fit linear mixed-effects models using the MLPE parameterization, which has been found to perform better than other regression-based statistical approaches^70^, to account for the non-independence of values within pairwise distance matrices and to distinguish the effects of multiple independent variables. Mixed-effects models were fit by maximum likelihood to test the effects of fixed factors (geographic, bioclimatic, and two resistance distances) with the random effect of populations. AIC and BIC were used as the objective criteria to evaluate model fit from four models of single fixed factor, six combinations of double fixed factors, four combinations of triple fixed factors, and the full model (the combinations of all fixed factors, Table 5).

Since IBE was suggested as the first-ranked model by both the partial Mantel test and model selection for MLPE (see Results), we further identified the most crucial environmental factors affecting genetic distance using two strategies. First, we re-executed the partial Mantel test by calculating the distance of each environmental factor between populations. To avoid unnecessary weighting due to intercorrelations among bioclimatic variables, we used variance inflation factor (VIF) analysis to reduce multicollinearity^71^. We discarded variables with high VIF values (VIF > 10) and then calculated the distances of the retained bioclimatic variables among populations to test the IBE hypothesis (Fig. 6). This remaining factor is the most likely environmental factor affecting the population genetic structure of *A. mongolicus*. Testing one variable by a Mantel (or partial Mantel) test has been suggested to be more credible than testing multiple variables^28^. Second, we performed model selection to evaluate 19 models with every single bioclimatic distance as the fixed factor in MLPE. These single-bioclimate-distance IBE models were ranked by AIC, and the model with the lowest AIC was suggested as the best one for the prediction of population genetic structure.

## Supplementary information

Dataset1

## Data Availability

All genetic and environmental data used in this study are available in the Supplementary Data.
